# Supplementary material for: Structure of the LdcB LD-Carboxypeptidase Reveals the Molecular Basis of Peptidoglycan Recognition
Source: Structure. 2014 Jul 8;22(7):949–60. doi: 10.1016/j.str.2014.04.015 (PMC4087270; doi:10.1016/j.str.2014.04.015)
Supplement: Document S1. Figures S1–S7 [file mmc1.pdf]

Structure, Volume *22*

Supplemental Information

Structure of the LdcB LD-carboxypeptidase Reveals the Molecular Basis of Peptidoglycan Recognition

Christopher N. Hoyland, Christine Aldridge, Robert M. Cleverley, Marie-Clémence Duchêne, George Minasov, Olena Onopriyenko, Karzan Sidiq, Peter J. Stogios, Wayne F. Anderson, Richard A. Daniel, Alexei Savchenko, Waldemar Vollmer, and Richard J. Lewis

Supplementary Figures

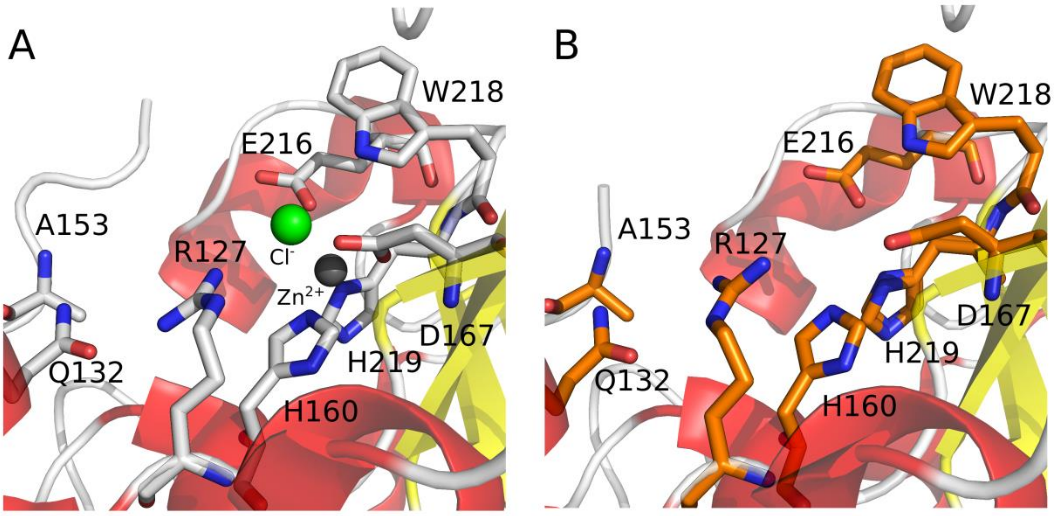

Fig. S1, related to Fig. 3. Comparison between apo and zinc-bound *Ba*LdcB.

The active sites of zinc-bound (A) and apo (B) *Ba*LdcB are shown in "stick" format, with a semi-transparent cartoon representation in the background. There are no major conformational changes in the absence of bound metal ion.

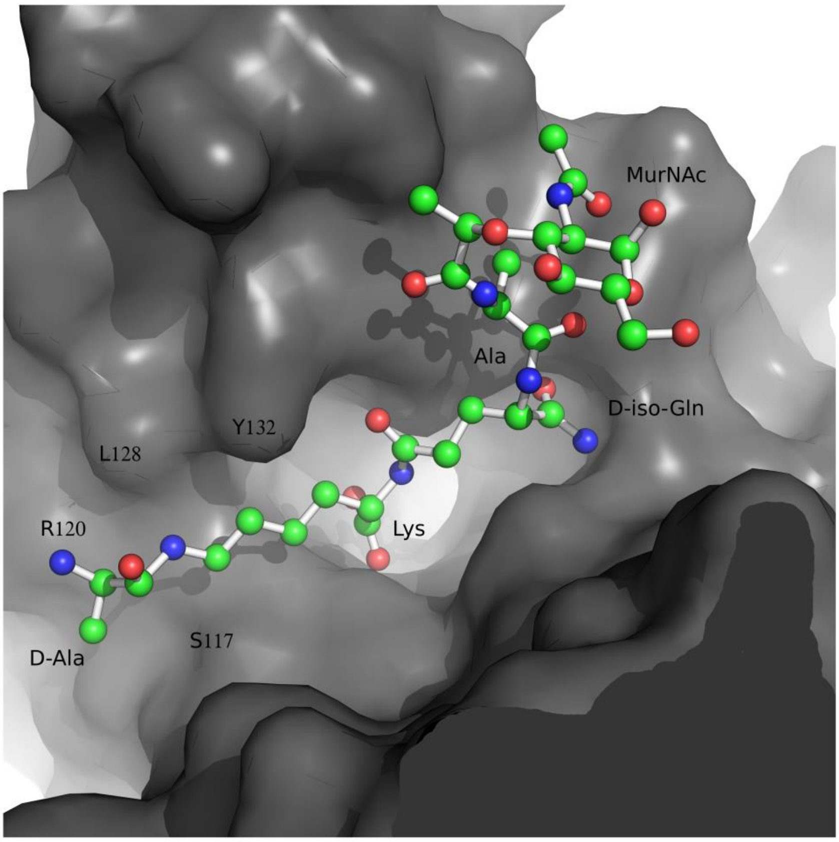

Fig. S2, related to Fig. 6. Proposed path of cross-linked cell wall peptides.

The terminal D-Asn of the ligand when bound to *Sp*LdcB occupies the S_1' subsite rather than projecting to the surface of the protein. Simple rotation of the lysyl's torsion angles presents the terminal D-aspargine to the surface of the protein to mimic the L-Lys-D-Ala (or L-Lys-Gly$_n$) cross-link observed between stem peptides. The path of the lysine is flanked by two highly conserved amino acids in LdcB sequences, Arg120 and Ser117, in addition to the functionally conserved Leu128. Tyr132 also flanks the lysine, but this amino acid is not conserved between sequences.

Supplementary Video

Video S1, related to Fig. 8. Conformational changes in SpLdcB on ligand binding.

Morph interpolation (UCSF Chimera) of the structural changes that occur upon ligand binding in *Sp*LdcB. α-helices are shown in red, β-strands in yellow and loops between secondary structure elements are coloured silver. The bound zinc ion is shown as a grey sphere. Key residues that move upon ligand binding are shown in stick representation and the movie was rendered in PyMOL.
