# Supplementary figures and images for: Structure of the LdcB LD-Carboxypeptidase Reveals the Molecular Basis of Peptidoglycan Recognition
Source: Structure. 2014 Jul 8;22(7):949–60. doi: 10.1016/j.str.2014.04.015 (PMC4087270; doi:10.1016/j.str.2014.04.015)

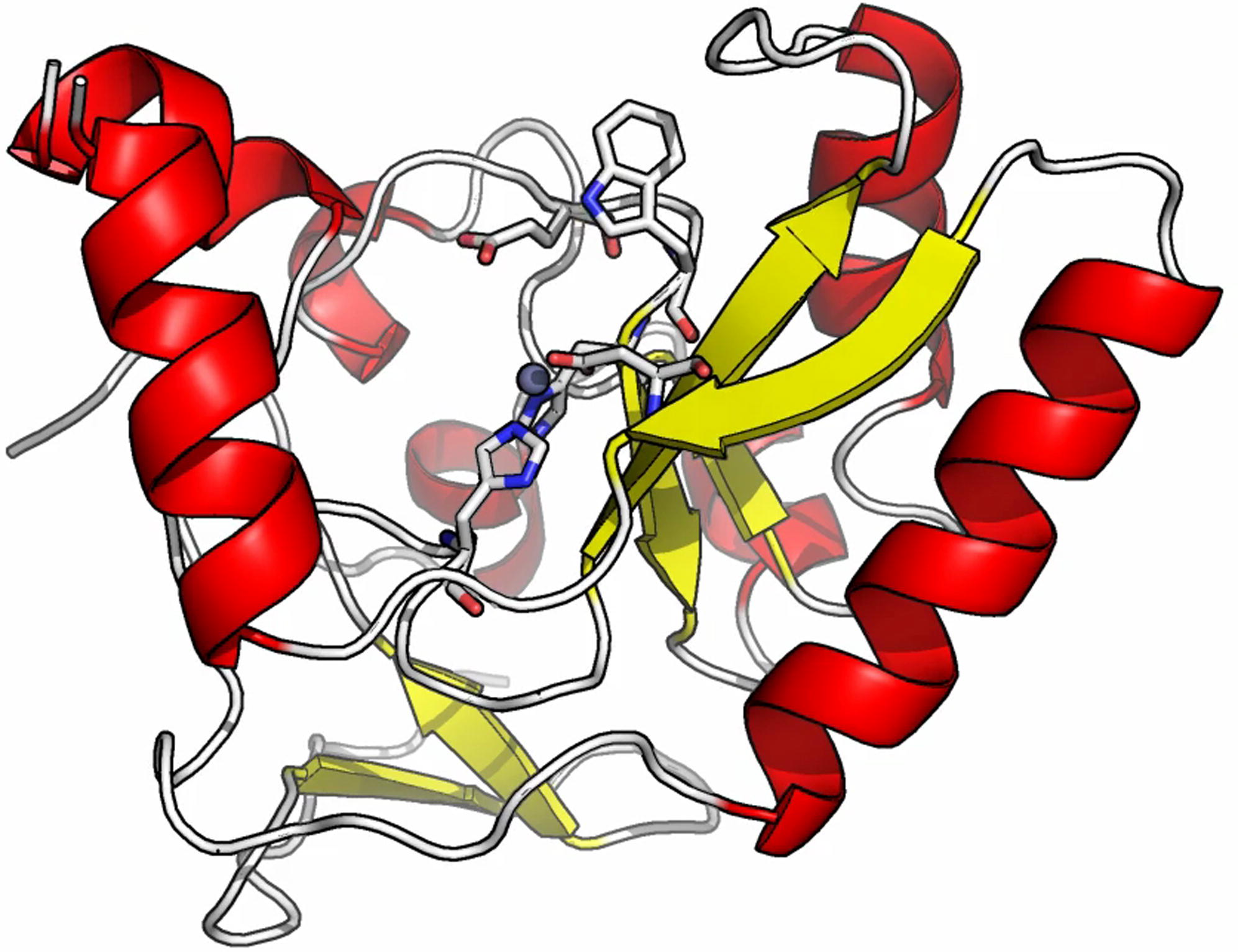

Supplement: Movie S1. Conformational Changes in SpLdcB on Ligand Binding, Related to Figure 7 — Morph interpolation (UCSF Chimera) of the structural changes that occur upon ligand binding in SpLdcB. α-helices are shown in red, β-strands are in yellow, and loops between secondary structure elements are colored silver. The bound zinc ion is shown as a grey sphere. Key residues that move on ligand binding are shown in stick representation, and the movie was rendered in PyMOL. [file mmc2.jpg]
