# Supplementary material for: Structure of the LdcB LD-Carboxypeptidase Reveals the Molecular Basis of Peptidoglycan Recognition
Source: Structure. 2014 Jul 8;22(7):949–60. doi: 10.1016/j.str.2014.04.015 (PMC4087270; doi:10.1016/j.str.2014.04.015)
Supplement: Document S2. Article plus Supplemental Information [file mmc3.pdf]

Structure
Article

Structure of the LdcB LD-Carboxypeptidase Reveals the Molecular Basis of Peptidoglycan Recognition

Christopher N. Hoyland,[1] Christine Aldridge,[2] Robert M. Cleverley,[1] Marie-Clémence Duchêne,[3] George Minasov,[4,5] Olena Onopriyenko,[5,6] Karzan Sidiq,[2] Peter J. Stogios,[5,6] Wayne F. Anderson,[4,5] Richard A. Daniel,[2] Alexei Savchenko,[5,6] Waldemar Vollmer,[2] and Richard J. Lewis[1,*]
[1]Institute for Cell and Molecular Biosciences, Newcastle University, Newcastle upon Tyne NE2 4HH, UK
[2]Centre for Bacterial Cell Biology, Institute for Cell and Molecular Biosciences, Newcastle University, Newcastle upon Tyne NE2 4AX, UK
[3]Institut des Sciences de la Vie, Université Catholique de Louvain, 1348 Louvain-la-Neuve, Belgium
[4]Department of Molecular Pharmacology and Biological Chemistry, Feinberg School of Medicine, Northwestern University, Chicago, IL 60611, USA
[5]Center for Structural Genomics of Infectious Diseases (CSGID)
[6]Department of Chemical Engineering and Applied Chemistry, 200 College Street, University of Toronto, Toronto, ON M5G 1L6, Canada
*Correspondence: r.lewis@ncl.ac.uk
http://dx.doi.org/10.1016/j.str.2014.04.015
This is an open access article under the CC BY license (http://creativecommons.org/licenses/by/3.0/).

SUMMARY

Peptidoglycan surrounds the bacterial cytoplasmic membrane to protect the cell against osmolysis. The biosynthesis of peptidoglycan, made of glycan strands crosslinked by short peptides, is the target of antibiotics like β-lactams and glycopeptides. Nascent peptidoglycan contains pentapeptides that are trimmed by carboxypeptidases to tetra- and tripeptides. The well-characterized DD-carboxypeptidases hydrolyze the terminal D-alanine from the stem pentapeptide to produce a tetrapeptide. However, few LD-carboxypeptidases that produce tripeptides have been identified, and nothing is known about substrate specificity in these enzymes. We report biochemical properties and crystal structures of the LD-carboxypeptidases LdcB from *Streptococcus pneumoniae*, *Bacillus anthracis*, and *Bacillus subtilis*. The enzymes are active against bacterial cell wall tetrapeptides and adopt a zinc-carboxypeptidase fold characteristic of the LAS superfamily. We have also solved the structure of *S. pneumoniae* LdcB with a product mimic, elucidating the residues essential for peptidoglycan recognition and the conformational changes that occur on ligand binding.

INTRODUCTION

Bacteria surround their cytoplasmic membrane with a large, semipermeable barrier, the peptidoglycan (PG) sacculus, which not only protects the cell from lysis from turgor but also helps to maintain cell shape. PG comprises repeating units of the *N*-acetylglucosamine-*N*-acetylmuramic acid (Glc*N*Ac—Mur*N*Ac) disaccharide that polymerizes into long glycan chains. Pentapeptides, usually comprising L-Ala—D-γ-Glu[Gln]—L-Lys—D-Ala—D-Ala, extend from the Mur*N*Ac moiety. In Gram-negative bacteria and some Gram-positive Firmicutes, the L-Lys at position 3 is replaced by *meso*-diaminopimelic acid (A_2pm), which is amidated in *Bacillus subtilis* (Vollmer et al., 2008a). Peptides protruding from adjacent glycan chains are crosslinked by transpeptidases to form the net-like PG sacculus, which the cell has to enlarge in order to grow and to divide (Sauvage et al., 2008). Newly inserted PG matures and is turned over by PG hydrolases, and the balance between synthesis and degradation of PG is key to normal cell division (Vollmer et al., 2008b). The importance of PG to the healthy physiology of a bacterium is highlighted by the fact that its biosynthesis is the target of some of our most successful antibiotics, β-lactams such as penicillin and glycopeptides such as vancomycin, resistance to which in pathogens is an urgent and currently unmet human health challenge.

In most species, the terminal D-alanine of the nascent pentapeptide stem is hydrolysed by DD-carboxypeptidases from the class C *p*enicillin *b*inding *p*rotein (PBP) family (Lovering et al., 2012). LD-carboxypeptidases subsequently hydrolyze the L-Lys[A_2pm]—D-Ala bond to release the tripeptide. Though tripeptides are found in many bacteria, very few of the corresponding LD-carboxypeptidases have been identified. Membrane preparations of *B. subtilis* have shown strong LD-carboxypeptidase activity against cell-wall-derived tetrapeptides (Arminjon et al., 1977), though the specific enzyme involved has not been isolated. The best characterized LD-carboxypeptidase is LD-carboxypeptidase A (LdcA), a cytoplasmic, penicillin-insensitive member of the S66 family of serine peptidases that is involved in PG recycling and is essential for cells entering the stationary phase (Metz et al., 1986; Templin et al., 1999). The cytoplasmic localization of LdcA precludes its involvement in the production of cell wall tripeptides. Other LD-carboxypeptidases either must be active within the periplasm of Gram-negative bacteria or are anchored to the exterior of the cell membrane in Gram-positives.

Several LD-carboxypeptidase genes have been identified recently, including *dacB* from *Lactococcus lactis* and *Streptococcus pneumoniae* (Barendt et al., 2011; Courtin et al., 2006), *csd6* from *Helicobacter pylori* (Sycuro et al., 2012) and *pgp2*

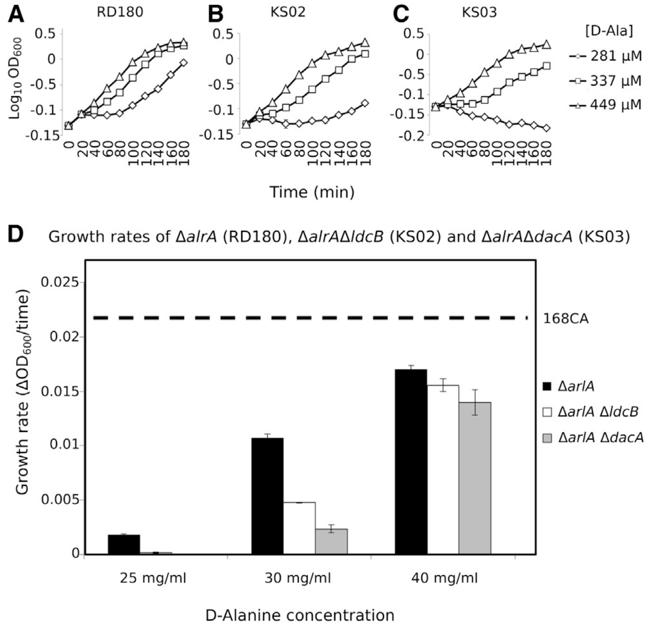

Figure 1. Effect of *dacA* and *ldcB* Alleles in a D-alanine Auxotroph

(A–C) The growth of strains (A) RD180 (Δ*alrA*), (B) KS02 (Δ*ldcB*Δ*alrA*), and (C) KS03 (Δ*dacA*Δ*alrA*) incubated at 37°C in LB medium supplemented with different concentrations of D-alanine was determined by monitoring the culture at OD_{600} and plotting it against time of incubation.

(D) The calculated growth rate of each strain at three different concentrations of exogenous D-alanine. The values shown represent the average of three independent experiments. The dashed line shows the growth rate of wild-type *B. subtilis* strain 168CA under the same conditions. The error bars represent the SD of growth rate means at a confidence level of 95%.

from *Campylobacter jejuni* (Frirdich et al., 2014), though it should be noted that *csd6* and *pgp2* share no sequence similarity with *dacB*. The corresponding null mutants exhibited increased amounts of tetrapeptides in the cell wall and a concomitant decrease in tripeptide content (Sycuro et al., 2012; Frirdich et al., 2014). The *S. pneumoniae dacB* mutant also displayed severe morphological defects, developing into small, spherical cells or elongating into long, thick cells, as a result of division asymmetry produced by random septum localization (Barendt et al., 2011). Both the *H. pylori csd6* and the *C. jejuni pgp2* mutants have a morphological defect, displaying a straight shape instead of the normal helical morphology, indicating that *csd6* and *pgp2* also contribute to cell shape maintenance (Frirdich et al., 2014; Sycuro et al., 2012). In *C. jejuni* (Frirdich et al., 2012) and *H. pylori* (Sycuro et al., 2010), cell shape is also linked to pathogenicity, and PG fragments released by growing cells are important signaling molecules for immune recognition and modulation in animals ranging from insects to man (Royet et al., 2011). Therefore, the PG plays an important role in bacterial shape maintenance, viability, virulence and immune signaling.

In this study, we have investigated the biochemical activity of *S. pneumoniae* and *B. subtilis* DacB against cell wall sacculi and synthetic tetrapeptides. Both proteins are shown to be active against cell-wall-derived tetrapeptides and synthetic tetrapeptides lacking the sugar moiety but are inactive against tetrapeptides terminating in L-alanine, confirming their classification as LD-carboxypeptidases. We therefore propose to rename these enzymes LD-carboxypeptidase B (LdcB). We present the crystal structures of both LdcB enzymes and the ortholog from *Bacillus anthracis*, all of which belong to the LAS (*l*ysostaphin, D-*A*la-D-Ala metallopeptidases, *s*onic hedgehog) family of zinc-dependent peptidases. The structure of *S. pneumoniae* LdcB with Mur*N*Ac—L-Ala—D-γ-Gln—L-Lys—(D-Asn) bound reveals the molecular basis of PG recognition and the key roles played in catalysis by active site elements. It is surprising that the active site is reminiscent, yet reversed, of that found in pancreatic carboxypeptidase A, suggesting that the enzymes have converged on a common catalytic mechanism.

RESULTS

Role for LdcB In Vivo

The mature PG of *S. pneumoniae* and *B. subtilis* contains mostly tripeptides, indicating the presence of PG-active peptidases in these organisms (Atrih et al., 1999; Bui et al., 2012). Both species contain DacA orthologs, DD-carboxypeptidases that cleave pentapeptides to tetrapeptides; however, the LD-carboxypeptidases that produce tripeptides from the tetrapeptides have remained elusive until recently. The PG of a *S. pneumoniae dacB* mutant contained mainly tetrapeptides, suggesting strongly that *Sp*DacB is an LD-carboxypeptidase (hereinafter renamed as *Sp*LdcB) (Barendt et al., 2011). Single mutants of the *dacA* and *dacB* orthologs in *B. subtilis*, *dacA*, and *yodJ* (hereinafter renamed as *ldcB*), had no obvious phenotype during vegetative growth, indicating that the trimming of peptides or the released D-Ala is not required for growth under laboratory conditions (Atrih et al., 1999). To test whether the released D-Ala becomes important under D-Ala limiting conditions, we constructed *dacA* and *ldcB* null mutants in a *B. subtilis* strain auxotrophic for D-Ala (an alanine racemase [*alrA*] null mutant) and assayed growth in the presence of D-Ala. The D-Ala-dependent strain, RD180, required at least 450 μM D-Ala for normal growth, and it grew more slowly at lower D-Ala concentrations (Figure 1). However, strains also bearing either the *dacA* (KS03) or the *ldcB* (KS02) alleles exhibited even more severe growth rate dependencies on D-Ala, with the *dacA* mutant the most severely affected. These data suggest strongly that LdcB is an LD-carboxypeptidase that releases D-Ala from the cell wall, which can be used to support growth under D-Ala-limiting conditions.

To test directly whether LdcB functions as an LD-carboxypeptidase, we generated *S. pneumoniae* and *B. subtilis ldcB* mutants and isolated their cell wall PG. Muropeptides were released with cellosyl and analyzed by HPLC (Figures 2A and 2B). The *ldcB* mutant strains lacked tripeptides and contained mainly tetrapeptides in their PGs, consistent with previous work indicating that LdcB is the main enzyme trimming cell wall tetrapeptides (Barendt et al., 2011). To obtain more direct evidence for an enzymatic activity, purified, recombinant LdcB enzymes from *S. pneumoniae* and *B. subtilis* (termed *Sp*LdcB and *Bs*LdcB) were incubated with PG isolated from the respective *ldcB* mutant strains before digesting with cellosyl and analyzing the resulting muropeptides by HPLC (Figures 2A and 2B). Both *Sp*LdcB and *Bs*LdcB almost quantitatively digested PG tetrapeptides with a

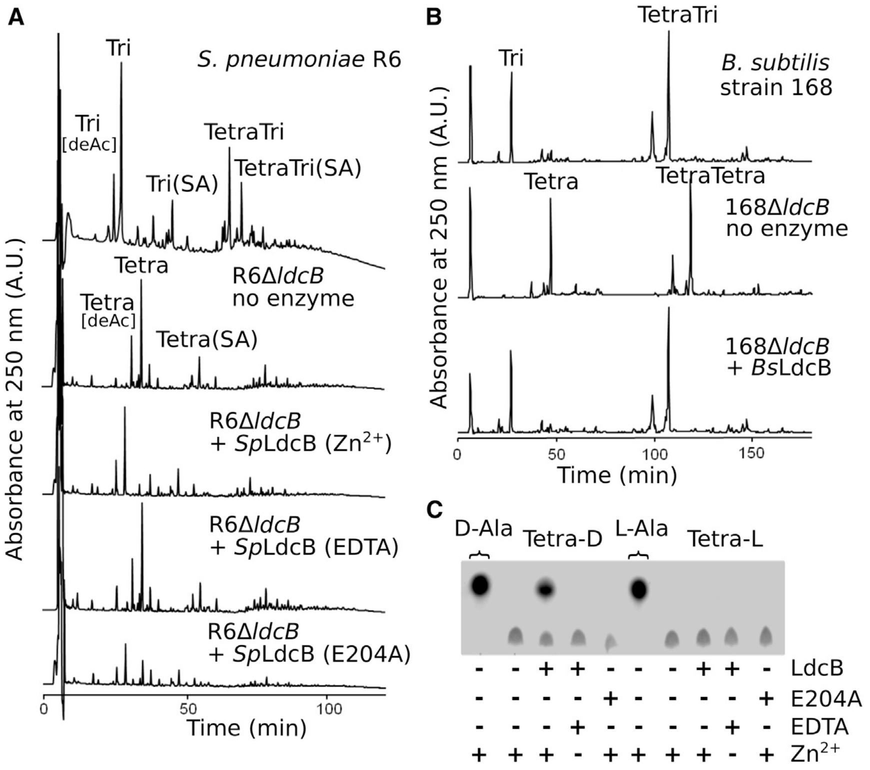

Figure 2. Activity of *Sp*LdcB and *Bs*LdcB against Peptidoglycan and Peptides

(A) HPLC chromatograms of muropeptides from *S. pneumoniae* strains, obtained after incubating PG with or without *Sp*LdcB or *Sp*LdcB(E204A) in the presence of Zn^{2+} or EDTA, followed by digestion with cellosyl and reduction with sodium borohydride. The muropeptides are: Tri, Glc*N*Ac—Mur*N*Ac(r)—L-Ala—D-γ-Gln—L-Lys; Tetra, Glc*N*Ac—Mur*N*Ac(r)—L-Ala—D-γ-Gln—L-Lys-D-Ala; TetraTri, Glc*N*Ac—Mur*N*Ac—L-Ala—D-γ-Gln—L-Lys—D-Ala—L-Lys—D-γ-Gln—L-Ala—Mur*N*Ac(r)—Glc*N*Ac; TetraTetra, Glc*N*Ac—Mur*N*Ac—L-Ala—D-γ-Gln—L-Lys—(D-Ala)—D-Ala—L-Lys—D-γ-Gln—L-Ala—Mur*N*Ac(r)—Glc*N*Ac; deAc, deacetylation at Glc*N*Ac; SA, L-Ser—L-Ala branch (at L-Lys). Glc*N*Ac, *N*-acetylglucosamine; Mur*N*Ac(r), *N*-acetylmuramitol.

(B) HPLC chromatograms of muropeptides from *B. subtilis* strains, obtained after incubating PG with or without *Bs*LdcB, followed by digestion with cellosyl and reduction with sodium borohydride. Muropeptides: Tri, Glc*N*Ac—Mur*N*Ac(r)—L-Ala—D-γ-Glu—*meso*-Dap(NH_2); Tetra, Glc*N*Ac—Mur*N*Ac(r)—L-Ala—D-γ-Glu—*meso*-Dap(NH_2)—D-Ala; TetraTri, Glc*N*Ac—Mur*N*Ac—L-Ala—D-γ-Glu—*meso*-Dap(NH_2)—D-Ala-*meso*-Dap(NH_2)— D-γ-Glu—L-Ala—Mur*N*Ac(r)—Glc*N*Ac; TetraTetra, Glc*N*Ac—Mur*N*Ac—L-Ala—D-γ-Glu—*meso*-Dap(NH_2)—(D-Ala)—D-Ala—*meso*-Dap(NH_2)— D-γ-Glu—L-Ala—Mur*N*Ac(r)—Glc*N*Ac; *meso*-Dap(NH_2), *meso*-diaminopimelic acid (amidated).

(C) TLC of *Sp*LdcB incubated with various substrates.

carboxy-terminal D-Ala producing the tripeptides, proving their LD-carboxypeptidase activity. *Sp*LdcB was active in the presence of Zn^{2+} ions and inactive in the presence of EDTA, suggesting a metal-ion-dependent peptidase mechanism.

SpLdcB and BsLdcB Are Catalytically Active against Synthetic Cell Wall Tetrapeptides

In addition to removing the terminal D-Ala from *S. pneumoniae* cell wall PG tetrapeptides (Figure 2A), *Sp*LdcB was also tested against synthetic tetrapeptides (L-Ala—D-Gln—L-Lys—D-Ala [tetra-D]) and its L-isomer, L-Ala—D-Gln—L-Lys—L-Ala [tetra-L]). Though these substrates lack the glycan component and differ further from the natural substrate by the replacement of D-γ-glutamine with D-glutamine, they have the potential to discriminate between LD- and LL-carboxypeptidase activities. *Sp*LdcB was active solely against the tetra-D ligand (Figure 2C), an activity that was dependent on the presence of zinc and abrogated totally in the presence of EDTA (Figure 2C).

Overall Structure of LdcB

The crystal structures of *Sp*LdcB and *Bacillus anthracis* LdcB (*Ba*LdcB) were solved by single wavelength anomalous dispersion (SAD); *Bs*LdcB was solved by molecular replacement. The LdcB enzymes are single domain proteins comprising two subdomains separated by a V-shaped cleft (Figures 3A–3C). The first subdomain comprises a four-stranded, antiparallel β sheet (β4–β7) that opposes the cleft, and the outside face of the β sheet is flanked by three α helices (α1, α4, and α6). The second subdomain comprises an N-terminal, three-stranded, antiparallel β sheet (β1–β3), somewhat discrete from the four helical bundle (α2, α3, α5, and α7) that makes up the rest of the subdomain. Though the *Ba*LdcB structure contains the same subdomain, it is less well structured in this region than *Bs*LdcB and *Sp*LdcB, and, as a consequence, the β1–β3 sheet is not formed. Neither subdomain is formed by a contiguous, linear part of the primary sequence; six loops (β3–α1, β4–α2, α3–β5, β6–α5, α5–β7, and α6–α7) connect the two subdomains.

The cleft between subdomains is 15–18 Å wide at the top and 15 Å deep and narrows down to the base where a Zn^{2+} ion, added to the enzyme during purification, is located (Figures 3A–3C), which is coordinated in a tetrahedral manner by three amino acids. In *Sp*LdcB, the liganding residues are His153, Asp160, and His207 (Figure 3D); the equivalents in *Bs*LdcB/*Ba*LdcB are His185/His160, Asp192/Asp167, and His244/His219 (Figures 3E and 3F). These zinc ligands are universally conserved in LdcB sequences, consistent with an important functional role in these enzymes. The active site of *Sp*LdcB contains unattributed residual electron density that occupies the position of the fourth ligand in the tetrahedral coordination sphere of the zinc (Figure 3D). To use the nomenclature common to proteases, the unattributed electron density extends as far as the S_1' subsite, the location ordinarily occupied by the scissile amino acid. The electron density in the S_1' subsite is of a size, shape, and location corresponding to that of alanine. The ligand is stabilized in this position by van der Waals' contacts between the alanyl methyl group

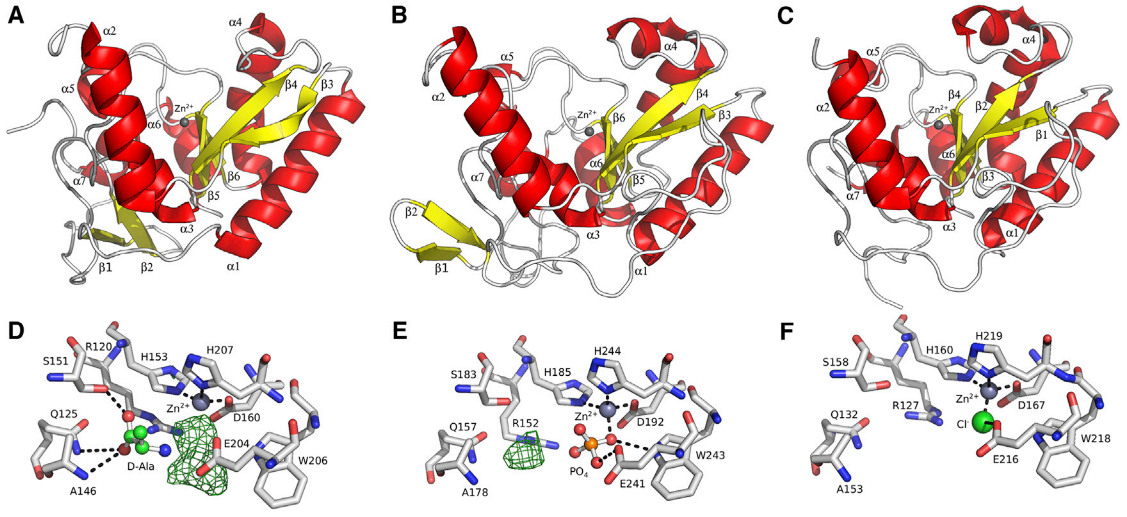

Figure 3. Structure of LdcB

(A–C) Cartoon representations of (A) *Sp*LdcB, (B) *Bs*LdcB, and (**C**) *Ba*LdcB; α helices (red) and β strands (yellow) are numbered. The loops between secondary structure elements are colored silver. The bound zinc ion is shown as a gray sphere.

(D–F) The active sites of (D) *Sp*LdcB, (E) *Bs*LdcB, and (F) *Ba*LdcB. A zinc ion is coordinated in a tetrahedral manner in the structures by two invariant histidines and a well-conserved aspartate. Residual electron density in *Sp*LdcB, shown in (D), corresponds to D-alanine in the S_1' subsite, and an unknown compound in S_1, adjacent to the zinc ion. Electron density corresponding to a phosphate ion, a component of the crystallization medium, in the S_1 subsite of *Bs*LdcB shown in (E) is a mimic of the tetrahedral transition state. A second feature in the S_1' subsite is unmodeled. The residual difference density features are shown contoured at a level of 3σ and hydrogen bonds are drawn as dashed black lines.

and the phenolic side chains of Tyr191 and Tyr201 and by hydrogen bonds to the side chains of Glu204 and the main chain amide nitrogen of Tyr144. Despite the high resolution of the *Sp*LdcB diffraction data, it was not possible to discriminate between either alanyl stereoisomer, both of which are present in the crystallization conditions. We have modeled D-Ala because it is the enzymatic product of these enzymes. However, residual electron density adjacent to the zinc ion could not be modeled satisfactorily with any ligand arising from the enzymatic activity, the crystallization media, or purification media; consequently, the remaining electron density has been left unmodeled. Mass differences between native and denatured protein samples also failed to identify candidate molecules corresponding to the residual electron density. Residual electron density in the vicinity of the fourth zinc ligand in *Bs*LdcB was fulfilled by a phosphate anion, a component of the crystallization conditions (Figure 3E), which refined satisfactorily. The phosphate is located so as to mimic the tetrahedral transition state of amide bond hydrolysis. There is a further unmodeled electron density feature (Figure 3E), this time in the S_1' subsite, that we have not been able to identify unambiguously.

Comparison of LdcB Structures

*Sp*LdcB, *Ba*LdcB, and *Bs*LdcB share pairwise sequence identities of ~30%, and they superimpose on ~155 common C_α atoms with a root-mean-square deviation (rmsd) of 1.5 Å–1.8 Å (Figures 3A–3C). The major differences between the LdcB structures are limited to the rather flexible and unconstrained N- and C-termini and the occasional longer loop in one structure in comparison to the other, as a direct consequence of sequence insertions. The active sites of *Sp*LdcB, *Ba*LdcB, and *Bs*LdcB are, to all extents and purposes, indistinguishable (Figures 3D–3F). A further *Ba*LdcB structure has been solved that contains no bound zinc ion, but there are negligible differences between it and its counterpart with zinc bound (Figure S1 available online). The active site of the LdcB enzymes is, therefore, sculpted in a robust fashion, competent for peptidolysis once the catalytic machinery is completed by the incorporation of zinc.

Structural Comparison with Other Peptidases

Using *Sp*LdcB as a search model in DALI (Holm and Rosenström, 2010), over 100 significantly similar structures were identified in the Protein Data Bank (PDB), including the structure of $VanXY_G$, a dipeptidase/DD-carboxypeptidase (PDB ID 4f78 [Meziane-Cherif et al., 2014]; DALI Z score = 17.0); an endolysin from the *Listeria* bacteriophage A500 (PDB ID 2vo9 [Korndörfer et al., 2008]; Z = 9.5); VanX, a DD-dipeptidase (PDB ID 1r44 [Bussiere et al., 1998]; Z = 6.6); and the N-terminal domain of sonic hedgehog, a protease involved in embryogenesis (PDB ID 2wg4 [Bishop et al., 2009]; Z = 5.3). Equivalent results were obtained using the structures of *Ba*LdcB and *Bs*LdcB as queries in DALI. The matched structures are all members of the wider LAS superfamily of proteases (Bochtler et al., 2004), which, although they share very little sequence identity, possess markedly similar active sites (Figure 4). By comparison with the $VanXY_G$ structure (Meziane-Cherif et al., 2014), the active site of the LdcB proteins is open and more solvent accessible. The open nature of the active site is a direct consequence of the absence of the β4-α5/bisubstrate selectivity loop in LdcB, which enables the recognition of PG, a molecule far greater in size than the dipeptides utilized by $VanXY_G$ (Meziane-Cherif et al., 2014).

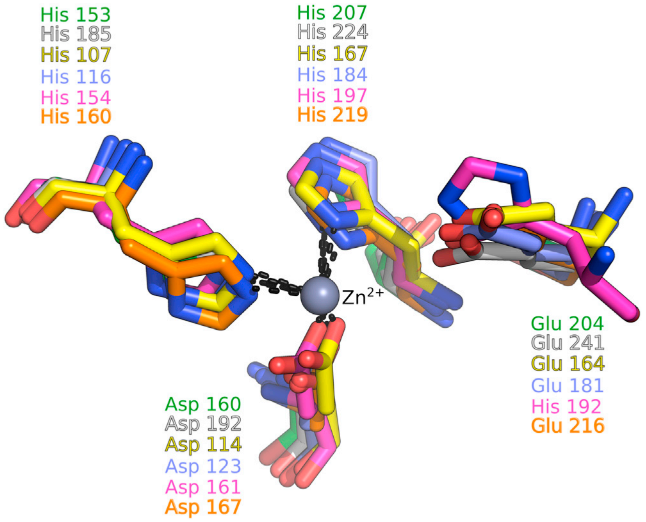

Figure 4. A Comparison of the Active Sites of LdcB and Other LAS Family Members

LdcB shares an active site closely superimposable on those of other LAS family enzymes. The zinc is coordinated by a H-$X_{(3-6)}$-D-$X_{(not\ conserved)}$-H motif. A glutamate that plays a key role in catalysis is also shown; note that, in D-Ala-D-Ala carboxypeptidase from *Streptomyces albus*, the equivalent amino acid is histidine, the first amino acid in the H-X-H motif common to most LAS carboxypeptidases. The structures shown, distinguished by the coloring of carbon atoms, are *Sp*LdcB (white), *Bs*LdcB (green), *Ba*LdcB (orange), *Enterococcus faecalis* D,D-dipeptidase/D,D-carboxypeptidase $VanXY_G$ (yellow; PDB ID 4f78), *Enterococcus faecium* aminodipeptidase VanX (blue; PDB ID 1r44) and *S. albus* D-Ala-D-Ala carboxypeptidase (pink; PDB ID 1lbu).

As with other LAS superfamily members, LdcB contains the characteristic zinc binding motif (Figure 4), with a consensus sequence of H-$X_{(3-6)}$-D-$X_{(not\ conserved)}$-H. The fourth zinc ligand in the structures of other LAS family members tends to be a solute from the crystallization milieu or a water that is likely to act as the nucleophile in the reaction (Bussiere et al., 1998). A second conserved motif in the LAS superfamily, H-X-H, of which the first histidine is the catalytic residue (Bochtler et al., 2004), is missing from LdcB enzymes; the catalytic histidine is replaced spatially by glutamate (i.e., Glu204 in *Sp*LdcB).

SpLdcB Cocrystallized with a Reaction Product

Despite attempting to crystallise LdcB enzymes with a range of synthetic ligands, one crystal structure was obtained, that of *Sp*LdcB in complex with Mur*N*Ac—L-Ala—D-γ-Gln—L-Lys—(D-Asn) (Figure 5A). This ligand is a close chemical analog of the true reaction product, as it contains half of the repeating unit of the glycan component of PG and comprises a tripeptide stem peptide that is branched at the L-lysine, the location of the crosslink position in natural PG (Figures 5F and 5G). There are two chemical differences between this synthetic mimic and the true reaction product. First, the ligand lacks Glc*N*Ac, the second sugar moiety of the glycan repeating unit in PG. Second, the terminal D-Asn group is covalently attached by a peptide bond between the main chain carbonyl of the terminal D-Asn and the side chain N_ζ of the penultimate lysine. In crosslinks between peptide stems, the side chain N_ζ of the penultimate lysine is linked by a peptide bond to the main chain carbonyl of a D-Ala. Hence, the L-Lys—(D-Asn) linkage is a faithful mimic of the natural L-Lys—(D-Ala), albeit the chemistry of the side chains of the terminal D-amino acids clearly differ.

There are six molecules of *Sp*LdcB in this crystallographic asymmetric unit, though none of the interfaces represent a stable multimeric form. Five of six protein chains could be built and refined satisfactorily; the electron density for the sixth copy could not be improved by any procedure to permit its reliable modeling, and its absence from the model contributes directly to the slightly inflated final R_{work} and R_{free} values (Table 1). The five built molecules are highly superimposable, with each other and with the apo *Sp*LdcB structure (rmsds between 0.5 Å and 1.1 Å; Table 2). In chains B, C, and E, the zinc is coordinated by a likely nucleophilic water to form tetrahedral coordination geometry. In chain D, the fourth zinc ligand is not evident, perhaps due to local disorder. Residual electron density was retained throughout refinement in only one of the five molecules, chain A, into which Mur*N*Ac—L-Ala—D-γ-Gln—L-Lys—(D-Asn) could be built and refined unambiguously (Figure 5A).

The structure of the ligand-bound form of *Sp*LdcB is entirely consistent with its LD-carboxypeptidase function. First, the Mur*N*Ac moiety is situated at the very top of the intersubdomain cavity and makes little contact with the protein. This part of the ligand has the highest B factors (a mean of 93 $Å^3$, versus a mean of 83 $Å^3$ for the intact ligand and a mean of 45 $Å^3$ for all atoms modeled) and, thus, the weakest electron density. The Mur*N*Ac O1 and O4 atoms, which define the location of the flanking β1- to β4-linked Glc*N*Ac sugars in PG, project away from the protein surface so that the glycan chain would be accommodated by the protein without steric clashes. The *N*-acetyl group at C1 is stabilized against the side chain of Met202 by van der Waals' forces (Figure 5B).

The C_α and C_β atoms of L-alanine at position 1 of the peptide pack against the face of Tyr144, a conserved amino acid in many LdcB sequences, to discriminate against the D-stereoisomer in this subsite, S_3. L-alanine is favored by contacts with Tyr144 and Met202, which discriminate against all L-amino acids bulkier than alanine (Figure 5B). The NH2 group of the D-γ-Gln of position 2 is located in subsite S_2, 2.3 Å away from and in hydrogen bonding distance of Glu204 (Figure 5C), an invariant amino acid in LdcB sequences. Stereoselectivity at this position is maintained because L-γ-glutamine would clash with Tyr144. The γ-glutamine is selected for by specific interactions with the main chain carbonyl of Ala203 and the side chain of Glu204 while ensuring that the scissile peptide bond is in the catalytic position. These key aspects of molecular recognition could not occur with commonly occurring amino acids. Canonical glutamine can be accommodated at this position in the synthetic peptide, tetra-D, because the absence of contacts with one face of the peptide provides scope for the conformational changes needed to accommodate glutamine in the absence of the glycan strands of the natural substrate.

Position 3 of the peptide is occupied by L-lysine. D-lysine could not be accommodated at this position because of the presence nearby of Glu107. As expected of a reaction product complex, the lysine carboxylate is located adjacent to the active site zinc in subsite S_1 (Figure 5D). One of the carboxylic oxygens completes the zinc tetrahedral geometry with His153/Asp160/His207, and the second oxygen forms a salt bridge with

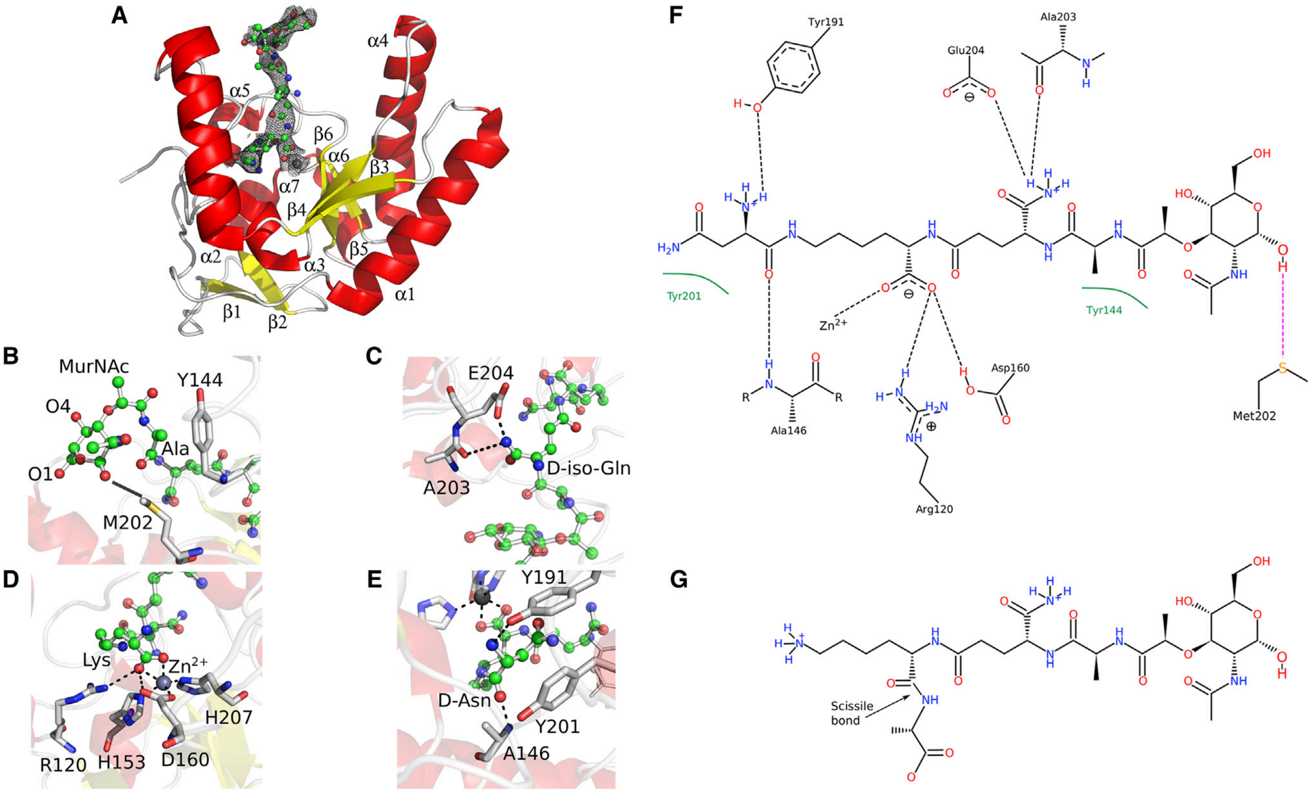

Figure 5. *Sp*LdcB with Bound Mur*N*Ac—L-Ala—D-γ-Gln—L-Lys—(D-Asn)

(A) Cartoon representation of *Sp*LdcB, drawn using the same color scheme as in Figure 3. The Mur*N*Ac—Ala—D-γ-Gln—L-Lys—(D-Asn) is drawn as a ball-and-stick model with Refmac-weighted $2F_{obs}$-F_{calc} electron density displayed at a contour level of 1 σ.

(B–E) The interactions made between the ligand and the protein at each subsite are shown successive panels: (B) S_3; (C) S_2; (D) S_1, and (E) S_1'. Key van der Waals' interactions are shown as a transparent silver line.

(F) A schematic of the interactions of *Sp*LdcB with the bound peptidoglycan mimic. Hydrogen-bond interactions are shown with a black dotted line, and van der Waals' forces are shown with a green arc.

(G) A schematic of the tetrapeptide substrate of *Sp*LdcB, drawn in the same manner as the peptidoglycan mimic in (F). The scissile bond is highlighted by an arrow. Crosslinks to other stem peptides would take place via the lysine's terminal amino group on the left.

Arg120, which is either arginine or lysine in LdcB orthologs. The L-lysine carboxylate thus represents a posthydrolysis state, placed appropriately for the peptide bond of the substrate to be hydrolyzed by the water molecule that fulfils the fourth zinc ligand in the absence of substrate.

The side chain of the L-lysine twists back against the rest of the peptide and projects into the protein core to place the terminal D-Asn branch in subsite S_1', against the main chain atoms of Tyr144 and the side chains of Tyr191, Tyr201, and His207 (Figure 5E). The carbonyl of the L-Lys—(D-Asn) peptide bond is contacted by the side chain of Ser151. However, it should be noted that subsite S_1' would be occupied by the scissile D-alanine in the presence of the reaction substrate, and support for this hypothesis comes from the structure of $VanXY_G$ solved in complex with a phosphinate transition state mimic (Figure 6A) (PDB ID 4muq) (Meziane-Cherif et al., 2014). The phosphinate moiety is a transition state mimic of peptide bond hydrolysis because the reaction proceeds via a tetrahedral intermediate that phosphinate mimics. $VanXY_G$ superimposes on *Sp*LdcB with an rmsd of 1.6 Å on 144 matched C_αs, and the alanyl carboxylate moiety of the phosphinate is situated in an analogous position to that of the L-Lys—(D-Asn) peptide linkage observed in *Sp*LdcB (Figure 6A). The phosphate atoms of the phosphinate transition state mimic superimpose almost identically with the phosphate anion modeled adjacent to the zinc ion in the structure of *Bs*LdcB (Figure 3D). Other than Ser151, a role in determining specificity for the terminal D-alanine can be invoked for Gln125, and both these amino acids are strictly conserved. The amide nitrogen of Ala146 also participates in stereoselectivity, but as this is a main chain atom, it is perhaps no surprise that sequence conservation is not maintained at this position.

However, crosslinks between peptide stems cannot be accommodated in the observed conformation because of the burial of the branched amino acid in subsite S_1' (Figure 6B). Mere rotation of the side chain torsion angles of the L-lysine would result in a more thermodynamically favored extended conformation for this side chain to result in it passing beyond Arg120, Leu128, and Tyr132 to project beyond the surface of the protein (Figure 6B; Figure S2). In this conformation, crosslinked peptide stems attached to the terminal N_ζ would not be sterically occluded by the body of the protein, consistent with the observed cleavage of crosslinked peptides (Figure 2A). In

Table 1. Summary of X-Ray Data Collection and Refinement Statistics

Parameters	*Sp*LdcB	*Bs*LdcB	*Sp*LdcB with Ligand	*Ba*LdcB	*Ba*LdcB with Zinc
PDB ID	4OX5	4OX3	4OXD	4JID	4MPH
Data collection					
Space group	*I*222	$P2_12_12_1$	*C*2	*I*222	*I*222
Unit Cell Dimensions					
a, b, c (Å)	48.8, 60.4, 138.2	42.7, 53.7, 102.4	346.0, 42.5, 79.3	76.6, 113.0, 124.6	76.8, 112.3, 125.7
α, β, γ (°)	90, 90, 90	90, 90, 90	90, 93.1, 90	90, 90, 90	90, 90, 90
Wavelength (Å)	0.98	0.97	0.92	0.97856	1.27696
Resolution (Å)	20.0–1.80 (1.84–1.80)	47.6–1.70 (1.73–1.70)	47.8–2.80 (2.95–2.80)	29.7–2.30 (2.36–2.30)	28.3–2.03 (2.06–2.03)
Multiplicity	24.3 (14.2)	3.2 (1.7)	3.4 (3.4)	4.2 (3.6)	11.8 (9.9)
R_{merge}[a]	0.077 (0.108)	0.072 (0.235)	0.134 (0.635)	0.093 (0.542)	0.101 (0.511)
I/σI	34.4 (2.4)	11.9 (2.4)	7.8 (1.9)	13.5 (2.4)	32.1 (4.2)
Completeness (%)	98.6 (86.9)	95.7 (89.3)	98.3 (98.3)	98.3 (94.3)	99.8 (96.9)
Refinement					
Resolution (Å)	20.0–1.80	47.6–2.0	47.8–2.80	29.7–2.3	28.3–2.03
No. of reflections	25,656 (1,157)	35,526 (1,057)	47,327 (1,979)	22,730 (1,226)	67,494 (3,374)
R_{work}/R_{free}[b]	0.191/0.253	0.176/0.247	0.273/0.335	0.194/0.259	0.151/0.189
No. of Atoms					
Protein	1,476	1,753	7,014	3,040	2,970
No. of proteins/AU	1	1	6	2	2
Ion/ligand	3/22	1/5	21/51	-/4	2/12
Water	197	230	111	424	466
B Factors (Å^2)					
Protein	41.8	16.0	44.3	45.3	35.8
Ion/ligand	36.9/50.7	10.7/23.8	38.7/69.7	–/49.8	17.9/60.0
Water	48.2	31.6	28.7	43.6	46.2
Rmsd					
Bond lengths (Å)	0.018	0.015	0.005	0.013	0.0012
Bond angles (°)	1.96	1.76	0.84	1.36	0.991
Ramachandran					
Favored (%)	96.1	96.7	95.8	97.6	98.0
Allowed (%)	100	100	99.6	99.7	100

Highest resolution shell shown in parentheses. AU, asymmetric units.
[a]$R_{merge} = \Sigma_h\Sigma_i|I_i(h) - \langle I(h)\rangle|/\Sigma_h\Sigma_i I_i(h)$, where $I_i(h)$ and $\langle I(h)\rangle$ are the *i*th and mean measurement of the intensity of reflection *h*.
[b]$R_{work}/R_{free} = \Sigma|F_p^{obs} - F_p^{calc}|/\Sigma F_p^{obs}$, where F_p^{obs} and F_p^{calc} are the observed and calculated structure factor amplitudes, respectively.

some bacteria, the lysine is replaced with diaminopimelic acid, a carboxy derivative of lysine that is amidated in bacilli. While the amidocarboxy derivative could be stabilized in the observed conformation by a similar set of specific interactions, it would also sterically occlude crosslinked peptide stems. Diaminopimelic acid is thus likely to follow the same path predicted for lysine by forming a simple, extended conformation to project into solvent.

Several key structural changes occur on ligand binding in *Sp*LdcB (Figure 7; Movie S1). First and foremost, substrates are occluded in the empty form of the enzyme by a loop between residues Gly163 and Glu171, which blocks access to the active site. Most of these residues (Asp167 to Glu171) move by 10–15 Å in the presence of ligand to open up the cleft by refolding into an additional turn at the N terminus of α-helix 4. The rearrangement of these residues is accompanied by further conformational changes in Trp206, the indole ring of which moves by up to 9 Å, which not only aids to open up the active site of the enzyme but also forces the side chain of Glu204 to rearrange to take up a position capable of interacting with the substrate. The importance of Glu204 and the zinc ion for catalysis is highlighted by the observation that tetra-D is a much poorer substrate for the E204A variant of *Sp*LdcB (Figure 2A) and by the absence of activity in the presence of EDTA (Figures 2A and 2C). The conformational changes seen on ligand binding to *Sp*LdcB contrasts with the absence of significant conformational changes described for $VanXY_G$ in complex with phosphinate (PDB ID 4muq) (Meziane-Cherif et al., 2014). Like LdcB, $VanXY_G$ maintains the H-$X_{(3–6)}$-D-$X_{(not\ conserved)}$-H motif, and as also observed in LdcB, the catalytic H-X-H motif is replaced by a glutamate. The resculpting of the N terminus of α-helix 4 in *Sp*LdcB to accommodate the tetrapeptide is not necessary in $VanXY_G$, because this enzyme hydrolyzes a much smaller substrate, D-Ala-D-Ala dipeptide, which is accommodated by the enzyme without significant rearrangement.

Table 2. Rmsd Values between Ligand-Bound *Sp*LdcB Chains (4OXD) and apo *Sp*LdcB (4OX5)

4OX5 and 4OXD Chain	4OX5	4OXD Chain A	4OXD Chain B	4OXD Chain C	4OXD Chain D	4OXD Chain E
4OX5	–	0.73	0.57	0.66	0.69	0.80
4OXD chain A	0.73	–	0.63	0.64	0.76	1.03
4OXD chain B	0.57	0.63	–	0.56	0.65	0.86
4OXD chain C	0.66	0.64	0.56	–	0.56	0.64
4OXD chain D	0.69	0.76	0.65	0.56	–	0.61
4OXD chain E	0.80	1.03	0.86	0.64	0.61	–

DISCUSSION

The presence of tripeptides in the bacterial cell wall implies that LD-carboxypeptidase activity must be present in the extracellular milieu. In this study, we have shown that LdcB, from both *S. pneumoniae* and *B subtilis*, are active against tetrapeptides found within the bacterial cell wall and are specific for LD linkages, confirming the LD-carboxypeptidase classification with a role in cell wall maturation. The precise in vivo role for LdcB remains to be determined; one possibility is that the enzyme allows recycling of D-Ala from the cell wall to support further cell wall synthesis and, thus, growth. Alternatively, the cleavage of D-Ala from the cell wall might permit the differentiation between old and new cell walls, a potentially powerful way to ensure the correct localization of new cell wall synthesis. The correct localization of new PG synthesis is likely to be more important to bacteria that have polarized cell growth, such as *Streptococci*, in comparison to those that have dispersed growth along the length of a cylinder (e.g., *Bacilli*) or that utilize a division-only mechanism, such as *Staphylococci*. The cell morphological defect observed in the *S. pneumoniae ldcB* mutant, and the absence of defect in the *B. subtilis ldcB* mutant, could be consistent with this premise. Further studies should distinguish these possibilities.

We also present crystal structures of *Ba*LdcB, *Bs*LdcB, and *Sp*LdcB, the latter also in complex with a PG-fragment analog that has enabled the identification of amino acids key to molecular recognition of the substrate and a potential tunnel through which crosslinked PG peptides may pass during proteolysis. LdcB belongs to the LAS group of zinc metalloproteases; several members of this family are active against PG, albeit with different substrate specificities to LdcB, including the Gly-Gly endopeptidase lysostaphin (Korndörfer et al., 2008) and the DD-/LD-endopeptidase MepA (Marcyjaniak et al., 2004). LdcB is dissimilar to other groups of LD-carboxypeptidases, e.g., *Pseudomonas aeruginosa* LD-carboxypeptidase LdcA (PDB ID 1ZRS) (Korza and Bochtler, 2005) that degrades cell wall tetrapeptides in the cytoplasm. LdcA utilizes a Ser-His-Glu catalytic triad in catalysis and belongs to the S66 serine protease family of enzymes, a group with which LdcB shares no similarity (Korza and Bochtler, 2005). Likewise, LdcB shares no sequence similarity with Csd6 and Pgp2, two recently discovered LD-carboxypeptidases that aid to maintain the helical shape of *H. pylori* (Sycuro et al., 2012) and *C. jejuni* (Frirdich et al., 2014). Csd6 and Pgp2 belong to an LD-transpeptidase family that utilizes a conserved Cys/His motif for catalysis (Biarrotte-Sorin et al., 2006), and are,

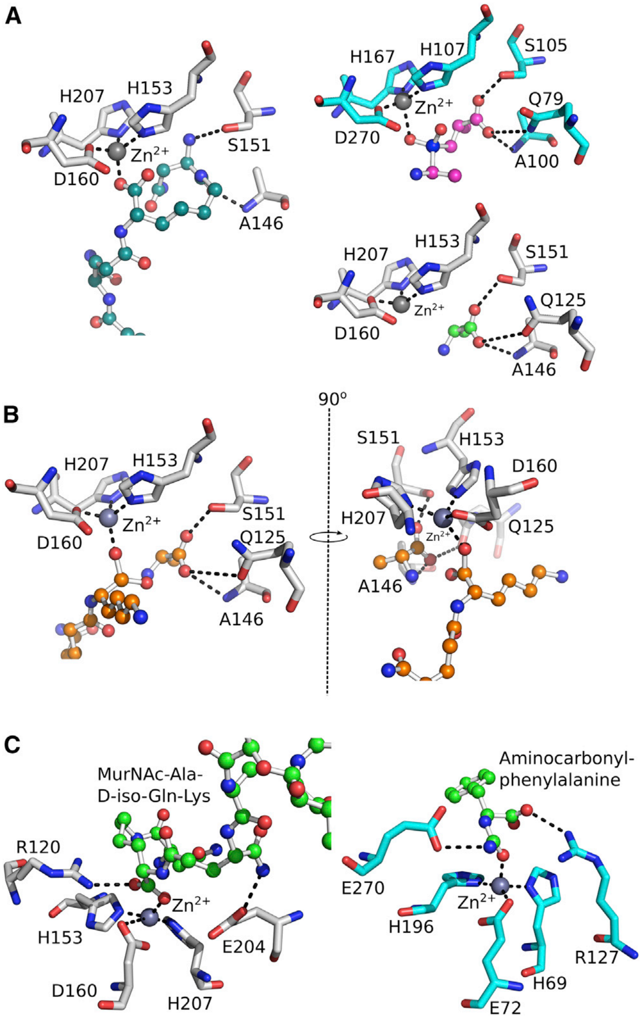

Figure 6. A Comparison of the Ligand-Bound Active Sites Carboxypeptidases

(A) The active site of *Sp*LdcB (white) with bound Mur*N*Ac—L-Ala-D-γ-Gln—L-Lys—(D-Asn) (turquoise carbons) is compared to that of VanXYg (blue) containing a phosphinate transition state analog (pink carbons) of D-Ala—D-Ala (PDB ID 4muq). The phosphinate in VanXYg coordinates the active site zinc in a similar manner to that of the lysyl carboxylate in the Mur*N*Ac—L-Ala-D-γ-Gln—L-Lys—(D-Asn) bound to *Sp*LdcB. The D-alanyl moiety of the phosphinate ligand in VanXYg, which occupies subsite S_1', is matched by the D-alanine (green carbons) in *Sp*LdcB (white) and the D-Asn of the Mur*N*Ac—L-Ala-D-γ-Gln—L-Lys—(D-Asn) ligand. The zinc ions are shown as gray spheres.

(B) The proposed positions of the two posthydrolysis ligands (orange carbons) in the active site of *Sp*LdcB (white). The D-alanine from the *Sp*LdcB structure occupies the S_1' site of the protein, while the lysine side chain has been rotated and modeled in an extended conformation. The altered conformation of the lysine presents the N_ζ to the surface of the protein, in an ideal position to accommodate peptide crosslinks. The zinc ions are shown as gray spheres.

(C) The active sites of *Sp*LdcB and pancreatic carboxypeptidase A (PDB ID 1hdu [Cho et al., 2002]) are compared. In each structure, the Zn^{2+} ion is coordinated by two histidines (His69 and His196 in carboxypeptidase A; His153 and His207 in *Sp*LdcB) and an acidic amino acid (Glu72 in carboxypeptidase A; Asp160 in *Sp*LdcB). The reversed locations of Arg127 and Glu270 in carboxypeptidase A relative to Arg120 and Glu204 in *Sp*LdcB is matched by a reversal of the path of the respective substrates through the active sites and the likely retention of a common catalytic mechanism.

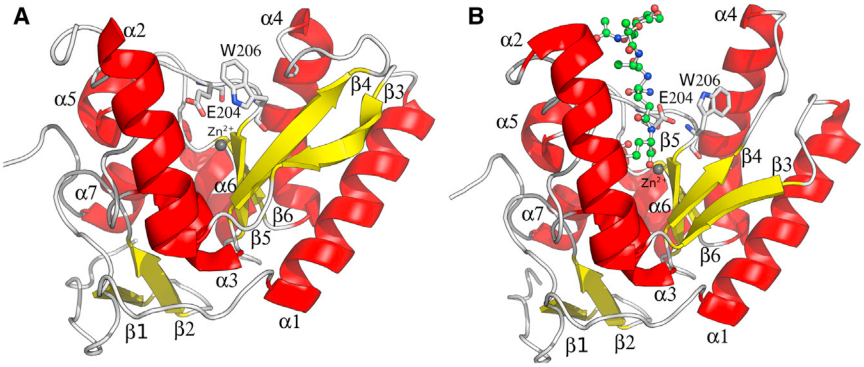

Figure 7. Conformational Changes in *Sp*LdcB on Ligand Binding

(A and B) Cartoon representations of (A) apo *Sp*LdcB and (B) *Sp*LdcB with bound Mur*N*Ac-Ala-D-γ-Gln-Lys-(D-Asn) using the same color scheme as in Figure 3. Key residues that move on ligand binding are shown in stick representation.

therefore, distinct from the metalloprotease LdcB. It is interesting that Csd6 can only hydrolyze D-Ala from monomeric stem peptides, whereas LdcB is active against both monomeric and crosslinked (tetra-tetra) stem peptides (Sycuro et al., 2013), but the absence of structural similarity between these enzymes precludes useful comparison.

While LdcB shares no structural homology with previously studied LD-carboxypeptidases, DALI searches revealed a strong similarity to members of the LAS superfamily. Of the two consensus sequences that characterize the LAS family, LdcB lacks the second His-rich motif, which is replaced spatially by the invariant Glu204, which is oriented away from the zinc ion. Given the residual activity of the E204A mutant (Figure 2A), Glu204 probably plays an indirect role in catalysis. The zinc ion probably plays a more direct role in catalysis, a hypothesis that is supported by the absence of enzymatic activity in the presence of EDTA (Figures 2A and 2C). Further support for the role of the metal ion, and the indirect role for the glutamate, comes from studies of other zinc-dependent metalloproteases such as pancreatic carboxypeptidase A, which is of historical importance given its role in the demonstration of Koshland's induced fit hypothesis (Christianson and Lipscomb, 1989; Koshland, 1958). Given the conformational changes seen on binding ligand (Figure 7; Movie S1), LdcB appears to be another example of induced fit. Though the two enzymes are structurally distinct, the active sites of LdcB and pancreatic carboxypeptidase A are, in essence, mirror images of one another. Both contain a zinc ion coordinated by two histidines (His69 and His196 in carboxypeptidase A; His153 and His207 in *Sp*LdcB) and an acidic amino acid (Glu72 in carboxypeptidase A; Asp160 in *Sp*LdcB). *Sp*LdcB Arg120 and Glu204 straddle the nucleophilic water, but their positions relative to their counterparts in pancreatic carboxypeptidase A (Arg127 and Glu270) are reversed (Figure 6C). Consequently, the path of the substrate through the active site is also reversed. Given the retention of the metal coordination and key adjacent amino acids in LdcB, it is entirely plausible that the reaction mechanism of LdcB occurs in a similar fashion to that of pancreatic carboxypeptidase A, utilizing the metal ion to activate the nucleophilic water to attack the scissile peptide bond. The enzymes would thus appear to have converged on a common mechanism, despite sharing little overall sequence and structural homology. Confirmation of the catalytic mechanism utilized by this enzyme, and its precise role in vivo, is the focus of current research activities in our laboratories.

EXPERIMENTAL PROCEDURES

Cloning

B. subtilis ldcB and *S. pneumoniae ldcB* were amplified by PCR from *B. subtilis* 168 and *S. pneumoniae* R6 genomic DNA, respectively, to generate fragments with NdeI and XhoI restriction sites. Both fragments were digested with NdeI and XhoI and ligated with NdeI/XhoI-digested pET28a. *B. anthracis ldcB* was PCR amplified from *B. anthracis* str. *Ames ancestor* genomic DNA and cloned into p15TV-LIC (Eschenfeldt et al., 2009) that codes for a N-terminal His_6 tag, followed by a tobacco etch virus (TEV) protease cleavage site. In all cases, the recombinant constructs were designed to remove membrane-anchoring amino acids to result in final clones encoding *Sp*LdcB amino acids 56–238; *Bs*LdcB, 61–273; and *Ba*LdcB, 55–243.

Strain Construction

S. pneumoniae R6Δ*ldcB* was constructed by transforming strain R6 with a PCR product covering the *ldcB*-deletion region from strain IU3880 (D39 Δ*cps2A*′(*cps2BCDETFG*)*H*′Δ*dacB* <> P_c-*kan-rpsL*) amplified with primers P136 and P137 (Barendt et al., 2011), followed by selection on tryptic soy broth blood agar plates containing 250 μg/ml kanamycin. All strains were grown in C + Y medium (Horne and Tomasz, 1993), containing kanamycin if required. Competent bacteria were obtained as described elsewhere, with the addition of competence-stimulating peptide (Vollmer and Tomasz, 2001).

B. subtilis strains were constructed by transformation of chromosomal DNA (Anagnostopoulos and Spizizen, 1961; Young and Spizizen, 1961) from strains bearing null mutations for *dacA* (MC01, *trpC2* Δ*dacA cat*) or *yodJ* (MC05, *trpC2* Δ*yodJ neo*) into strain RD180 (*trpC2* Δ*alrA zeo*), selecting for the appropriate resistance markers (chloramphenicol or kanamycin at 5 μg/ml) in the presence of 40 μg/ml D-alanine, yielding strains KS03 and KS02, respectively.

Growth Curves

B. subtilis strains RD180, KS02, and KS03 were grown overnight at 30°C in Luria broth (LB) supplemented with 40 μg/ml D-Ala. The overnight cultures were diluted 1:10 with LB supplemented with 40 μg/ml D-Ala and grown at 37°C for 2 hr. The cells were collected by centrifugation and resuspended in D-Ala-free media at 37°C. This suspension was used to inoculate media with various concentrations of D-Ala to an optical density 600 (OD_{600}) of 0.05, and the subsequent growth of each culture at 37°C was monitored by determining the optical density at 600 nm every 20 min for 3 hr. The growth rate was determined during the exponential growth phase only.

Mutagenesis

A modified QuikChange protocol, incorporating "touchdown" (Don et al., 1991), was used for site-directed mutagenesis. The annealing temperature of the first 10 cycles was reduced by one degree per cycle, from 68°C to 58°C, before a further ten cycles were performed at a constant annealing temperature of 58°C. The extension temperature was maintained at 72°C for 11 min per cycle. Mutagenic primers (0.3 μl) were mixed with 50 ng of template DNA, 0.5 mM dinucleotide triphosphates, and 2.5 U of *Pfu* DNA polymerase in a total reaction volume of 20 μl. The template plasmid was digested using *Dpn*I.

Peptidoglycan Activity Assays

Cell wall and PG from *S. pneumoniae* and *B. subtilis* strains were isolated by published procedures (Bui et al., 2012; Garcia-Bustos et al., 1988). To test for LD-carboxypeptidase activity, PG from *S. pneumoniae* R6Δ*ldcB*

(1.6 mg/ml) or *B. subtilis* 168Δ*ldcB* (3.1 mg/ml) was incubated in a total volume of 160 μl of 20 mM sodium phosphate, pH 4.8, with 0.13 mg/ml *Sp*LdcB and 0.31 mg/ml *Bs*LdcB, respectively, for 16 hr at 37°C. If required, 0.6 mM Zn^{2+} or 10 mM EDTA was added, and control samples contained no added enzyme. The PG was digested with 25 μg/ml of the muramidase cellosyl (a kind gift of Hoechst AG) for 24 hr at 37°C. The samples were boiled for 10 min and centrifuged for 10 min at 15,000 × *g*. The muropeptides present in the supernatant were reduced with sodium borohydride and analyzed by high-pressure liquid chromatography (HPLC) (Bui et al., 2012; Garcia-Bustos et al., 1988). *S. pneumoniae* and *B. subtilis* muropeptide peaks were assigned based on their published elution profile and retention times (Bui et al., 2012; Atrih et al., 1999). Fractions containing the reduced muropeptides Tetra and TetraTetra present in *B. subtilis* strain 168Δ*ldcB* were collected and analyzed by mass spectrometry (Bui et al., 2009). We determined a neutral mass of 940.4353 atomic mass units (amu) for Tetra (theoretical: 940.4237 amu) and of 1862.8406 amu for TetraTetra (theoretical: 1862.8368 amu).

Thin-Layer Chromatography

Tetrapeptides Ala-D-Gln-L-Lys-D-Ala (Tetra-D; Activotec) or Ala-D-Gln-L-Lys-L-Ala (Tetra-L; Activotec) at 20 mM were incubated with 700 uM of zinc-loaded *Sp*LdcB or *Sp*LdcB-E153A for 2 hr at 37°C in a buffer of 10 mM HEPES/NaOH, pH 8.0, 100 mM NaCl in a reaction volume of 10 μl. One sample of *Sp*LdcB contained 50 mM EDTA. Ethanol (5 μl) was added to the reactions before spotting on to silica gel thin-layer chromatography (TLC) aluminum foils (Sigma). The TLCs were developed in a running buffer of butan-1-ol, acetic acid, water in a 3:1:1 ratio for 40 min before staining with 0.1% ninhydrin solution in ethanol (Sigma). The TLC foils were dried and warmed with a domestic hairdryer for visualizing the results.

Protein Overexpression and Purification

*Bs*LdcB and *Sp*LdcB were prepared in the same way. The proteins were overexpressed in the methionine auxotrophic B834(DE3) strain of *E. coli* by growth in LB media (or in minimal medium supplemented with selenomethionine for selenomethionine-labeled protein) at 37°C up to an OD_{600} of 0.4 before induction with 1 mM IPTG for 18 hr at 16°C. Cells were harvested by centrifugation at 3000 *g* for 25 min at 4°C and the pellet was resuspended in 40 ml buffer A (50 mM HEPES.NaOH, pH 8.0, 300 mM NaCl, 20 mM imidazole) per liter of culture and lysed on ice for 3 min with a Sonopuls HD2070 (Bandelin) sonicator. The cell lysate was clarified at 33000 *g* for 20 min and the supernatant loaded onto a 5 ml Ni^{+}-NTA column (GE Healthcare) pre-equilibrated with buffer A. The column was washed with buffer A before elution of bound proteins with buffer B (50 mM HEPES/NaOH, pH 8.0, 300 mM NaCl, 500 mM imidazole). The fractions containing LdcB were pooled and the N-terminal hexahistidine tag was removed by digestion with thrombin (Sigma-Aldrich) at 20°C for 16 hr at a ratio of 1 unit of enzyme per mg of LdcB. LdcB was concentrated to a volume of 1 ml before loading onto a Superdex S75 HiLoad 16/60 (GE Healthcare) gel filtration column pre-equilibrated in buffer C (10 mM HEPES/NaOH, pH 8.0, 100 mM NaCl).

For *Ba*LdcB, freshly transformed *E. coli* BL21-CodonPlus (DE3)-RIPL cells were grown in 0.5 l of LB medium containing 100 μg/ml ampicillin. Cultures were grown at 37°C to an OD_{600} of 0.8 and induced for 16 hr at 16°C with 1 mM IPTG, and the cells were harvested by centrifugation and resuspended in 20 ml of buffer A (50 mM HEPES, pH 7.5, 300 mM NaCl, 10% [v/v] glycerol) supplemented with 10% (v/v) BugBuster 10× Protein Extraction Reagent (Novagen), 25 U Benzonase (Sigma-Aldrich), and 5 mM imidazole. The mixture was stirred for 10 min at room temperature and centrifuged at 20,000 × *g* for 45 min, and the supernatant was applied to a 1 ml HisTrap Fast Flow column (GE Healthcare) equilibrated with buffer A containing 40 mM imidazole. The protein was eluted with buffer A with a gradient of 40–500 mM imidazole over 20 ml. Fractions containing the recombinant His-tagged proteins were identified by SDS-PAGE; pooled; dialyzed overnight against 3 l of 50 mM HEPES, pH 7.5, 300 mM NaCl, 5% (v/v) glycerol, 1 mM TCEP; and stored at −80°C. The His_6-tag was cleaved by TEV protease in 500 mM NaCl, 50 mM Tris-HCl, pH 7.5, 1 mM TCEP, 1 mM EDTA, 20% glycerol.

Crystallization

*Bs*LdcB and *Sp*LdcB were concentrated to 15 mg/ml, and two molar equivalents of zinc chloride were added. Crystallization conditions for *Bs*LdcB and *Sp*LdcB were obtained by sitting drop vapor diffusion at 20°C. The best *Sp*LdcB crystals were obtained from a 0.1 M mixture of amino acids (glycine, L-glutamate, and racemates of alanine, serine, and lysine), 0.1 M Morpheus buffer system 2, pH 7.5, and 37.5% 2-Methyl-2,4-pentanediol (MPD)/polyethylene glycol (PEG) 1000/PEG 3350 (Gorrec, 2009). A single *Bs*LdcB crystal grew from 0.1 M phosphate/citrate buffer, pH 4.2, 40% PEG 300 over a period of ~2 months. Crystals of *Sp*LdcB with bound Mur*N*Ac—L-Ala—D-γ-Gln—L-Lys—(D-Asn) (InvivoGen) were obtained by incubating the protein with the ligand at 2 mM final concentration for 30 min at room temperature prior to crystallization against a mother liquor of 0.1 M ammonium chloride, 0.1 M HEPES/NaOH, pH 8.0, and 20% PEG 6000. Crystals of *Ba*LdcB (zinc free) were grown at 23°C using hanging-drop vapor diffusion by mixing 20 mg/ml protein with reservoir solution containing 0.3 M sodium chloride, 0.1 M HEPES, pH 7.5. As soaking of zinc chloride into *Ba*LdcB apo-protein crystals did not provide sufficient occupancy of zinc into the active site, crystals of *Ba*LdcB (zinc bound) were grown at 23°C by hanging-drop vapor diffusion by mixing 20 mg/ml protein and 5 mM zinc chloride prior to crystallization with 0.2 M ammonium acetate, 0.1 M sodium acetate, and 30% PEG 4000.

Data Collection and Processing

All crystals were cryoprotected with paratone-N before flash cooling in liquid nitrogen prior to data collection at 100 K. Diffraction data for *Bs*LdcB and *Sp*LdcB were on beamline IO2 of the Diamond Light Source. For selenomethionine-labeled *Sp*LdcB, diffraction data were collected at a wavelength of 0.98 Å to permit phasing by selenomethionine-SAD (Se-SAD). Diffraction data were integrated with XDS (Kabsch, 2010) and reduced with SCALA (Evans, 2006). For *Ba*LdcB, diffraction data were collected at on beamlines 21-ID-G at Life Sciences Collaborative Access Team, Advanced Photon Source, at 0.97856 Å (selenomethionine peak; zinc free) and 1.27696 Å (Zn^{2+} peak; zinc bound). The *Ba*LdcB diffraction data were integrated and reduced with HKL-3000 (Minor et al., 2006).

Structure Solution

The structure of *Sp*LdcB was solved using SHELX (Sheldrick, 2008) as implemented in the HKL2MAP interface (Pape and Schneider, 2004). An initial model was built by PHENIX.AUTOSOLVE (Adams et al., 2011). The completed *Sp*LdcB structure was used as the molecular replacement search model in PHASER (McCoy et al., 2007) to solve *Bs*LdcB and the *Sp*LdcB-ligand complex. The *Ba*LdcB Zn^{2+}-bound structure was solved by SAD phasing using PHENIX.solve (Adams et al., 2011), which identified the positions of the two bound Zn^{2+} ions. The occupancies of the zinc atoms were refined during model refinement. The *Ba*LdcB Zn^{2+}-free structure was solved by molecular replacement using the D,D-peptidase domain of the apo structure of $VanXY_G$ (PDB ID 4f78) (Meziane-Cherif et al., 2014).

For all structures, manual building cycles in COOT (Emsley et al., 2010) were interspersed with restrained refinement in PHENIX.REFINE (Afonine et al., 2012). B factors were refined isotropically for all structures apart from *Bs*LdcB, which was refined anisotropically. Noncrystallographic symmetry refinement was not used. Average B factor and geometric parameters were calculated using PHENIX and verified with PHENIX.refine and the Research Collaboratory for Structural Bioinformatics PDB Validation server. Data collection and final model refinement statistics for all structures can be found in Table 1. The graphics programs UCSF Chimera and PyMOL were used to generate all molecular figures presented.

ACCESSION NUMBERS

The PDB accession numbers for *Sp*LdCB, *Bs*LdcB, *Sp*LdcB with bound ligand, apo *Ba*LdcB, and *Ba*LdcB with bound zinc reported in this paper are 4OX5, 4OX3, 4OXD, 4JID, and 4MPH, respectively.

SUPPLEMENTAL INFORMATION

Supplemental Information includes seven figures and one movie and can be found with this article online at http://dx.doi.org/10.1016/j.str.2014.04.015.

ACKNOWLEDGMENTS

We thank the staff at the Diamond Synchrotron Light Source for access to, and help with, its beamlines for X-ray diffraction data collection. We thank Arnaud Baslé, Joseph Newman, and Vincent Rao for useful discussions; Atikah Mohd Sukor for excellent technical assistance; Z. Wawrzak, S. Shatsman, and S.N. Peterson for assistance with data collection and refinement; and T. Skarina for assistance with purification and crystallization. We also thank the authors of Meziane-Cherif et al. (2014) for sharing results and atomic coordinates prior to publication. Finally, we thank Joe Gray of the Newcastle University Pinnacle-Proteomics and Biological Mass Spectrometry facility for mass spectrometry analysis of muropeptide fractions. This project has been funded in whole or in part with federal funds from the National Institute of Allergy and Infectious Diseases, National Institutes of Health, U.S. Department of Health and Human Services (contracts HHSN272200700058C and HHSN2722012000026C), and by the UK Biotechnology and Biological Sciences Research Council in an award to R.A.D., W.V., and R.J.L. (BB/G015902/1).

Received: March 4, 2014
Revised: April 24, 2014
Accepted: April 29, 2014
Published: June 5, 2014

REFERENCES

Adams, P.D., Afonine, P.V., Bunkóczi, G., Chen, V.B., Echols, N., Headd, J.J., Hung, L.W., Jain, S., Kapral, G.J., Grosse Kunstleve, R.W., et al. (2011). The Phenix software for automated determination of macromolecular structures. Methods *55*, 94–106.

Afonine, P.V., Grosse-Kunstleve, R.W., Echols, N., Headd, J.J., Moriarty, N.W., Mustyakimov, M., Terwilliger, T.C., Urzhumtsev, A., Zwart, P.H., and Adams, P.D. (2012). Towards automated crystallographic structure refinement with phenix.refine. Acta Crystallogr. D Biol. Crystallogr. *68*, 352–367.

Anagnostopoulos, C., and Spizizen, J. (1961). Requirements for transformation in *Bacillus subtilis*. J. Bacteriol. *81*, 741–746.

Arminjon, F., Guinand, M., Vacheron, M.J., and Michel, G. (1977). Specificity profiles of the membrane-bound γ-D-glutamyl-(L)meso-diaminopimelateendopeptidase and LD-carboxypeptidase from *Bacillus sphaericus* 9602. Eur. J. Biochem. *73*, 557–565.

Atrih, A., Bacher, G., Allmaier, G., Williamson, M.P., and Foster, S.J. (1999). Analysis of peptidoglycan structure from vegetative cells of *Bacillus subtilis* 168 and role of PBP 5 in peptidoglycan maturation. J. Bacteriol. *181*, 3956–3966.

Barendt, S.M., Sham, L.T., and Winkler, M.E. (2011). Characterization of mutants deficient in the L,D-carboxypeptidase (DacB) and WalRK (VicRK) regulon, involved in peptidoglycan maturation of *Streptococcus pneumoniae* serotype 2 strain D39. J. Bacteriol. *193*, 2290–2300.

Biarrotte-Sorin, S., Hugonnet, J.E., Delfosse, V., Mainardi, J.L., Gutmann, L., Arthur, M., and Mayer, C. (2006). Crystal structure of a novel β-lactam-insensitive peptidoglycan transpeptidase. J. Mol. Biol. *359*, 533–538.

Bishop, B., Aricescu, A.R., Harlos, K., O'Callaghan, C.A., Jones, E.Y., and Siebold, C. (2009). Structural insights into hedgehog ligand sequestration by the human hedgehog-interacting protein HHIP. Nat. Struct. Mol. Biol. *16*, 698–703.

Bochtler, M., Odintsov, S.G., Marcyjaniak, M., and Sabala, I. (2004). Similar active sites in lysostaphins and D-Ala-D-Ala metallopeptidases. Protein Sci. *13*, 854–861.

Bui, N.K., Gray, J., Schwarz, H., Schumann, P., Blanot, D., and Vollmer, W. (2009). The peptidoglycan sacculus of *Myxococcus xanthus* has unusual structural features and is degraded during glycerol-induced myxospore development. J. Bacteriol. *191*, 494–505.

Bui, N.K., Eberhardt, A., Vollmer, D., Kern, T., Bougault, C., Tomasz, A., Simorre, J.P., and Vollmer, W. (2012). Isolation and analysis of cell wall components from *Streptococcus pneumoniae*. Anal. Biochem. *421*, 657–666.

Bussiere, D.E., Pratt, S.D., Katz, L., Severin, J.M., Holzman, T., and Park, C.H. (1998). The structure of VanX reveals a novel amino-dipeptidase involved in mediating transposon-based vancomycin resistance. Mol. Cell *2*, 75–84.

Cho, J.H., Kim, D.H., Chung, S.J., Ha, N.C., Oh, B.H., and Yong Choi, K. (2002). Insight into the stereochemistry in the inhibition of carboxypeptidase A with N-(hydroxyaminocarbonyl)phenylalanine: binding modes of an enantiomeric pair of the inhibitor to carboxypeptidase A. Bioorg. Med. Chem. *10*, 2015–2022.

Christianson, D.W., and Lipscomb, W.N. (1989). Carboxypeptidase A. Acc. Chem. Res. *22*, 62–69.

Courtin, P., Miranda, G., Guillot, A., Wessner, F., Mézange, C., Domakova, E., Kulakauskas, S., and Chapot-Chartier, M.P. (2006). Peptidoglycan structure analysis of *Lactococcus lactis* reveals the presence of an L,D-carboxypeptidase involved in peptidoglycan maturation. J. Bacteriol. *188*, 5293–5298.

Don, R.H., Cox, P.T., Wainwright, B.J., Baker, K., and Mattick, J.S. (1991). 'Touchdown' PCR to circumvent spurious priming during gene amplification. Nucleic Acids Res. *19*, 4008.

Emsley, P., Lohkamp, B., Scott, W.G., and Cowtan, K. (2010). Features and development of Coot. Acta Crystallogr. D Biol. Crystallogr. *66*, 486–501.

Eschenfeldt, W.H., Lucy, S., Millard, C.S., Joachimiak, A., and Mark, I.D. (2009). A family of LIC vectors for high-throughput cloning and purification of proteins. Methods Mol. Biol. *498*, 105–115.

Evans, P. (2006). Scaling and assessment of data quality. Acta Crystallogr. D Biol. Crystallogr. *62*, 72–82.

Frirdich, E., Biboy, J., Adams, C., Lee, J., Ellermeier, J., Gielda, L.D., Dirita, V.J., Girardin, S.E., Vollmer, W., and Gaynor, E.C. (2012). Peptidoglycan-modifying enzyme Pgp1 is required for helical cell shape and pathogenicity traits in *Campylobacter jejuni*. PLoS Pathog. *8*, e1002602.

Frirdich, E., Vermeulen, J., Biboy, J., Soares, F., Taveirne, M.E., Johnson, J.G., DiRita, V.J., Girardin, S.E., Vollmer, W., and Gaynor, E.C. (2014). Peptidoglycan LD-carboxypeptidase Pgp2 influences Campylobacter jejuni helical cell shape and pathogenic properties and provides the substrate for the DL-carboxypeptidase Pgp1. J. Biol. Chem. *289*, 8007–8018.

Garcia-Bustos, J.F., Chait, B.T., and Tomasz, A. (1988). Altered peptidoglycan structure in a pneumococcal transformant resistant to penicillin. J. Bacteriol. *170*, 2143–2147.

Gorrec, F. (2009). The MORPHEUS protein crystallization screen. J. Appl. Crystallogr. *42*, 1035–1042.

Holm, L., and Rosenström, P. (2010). Dali server: conservation mapping in 3D. Nucleic Acids Res. *38* (Web Server issue), W545–W549.

Horne, D.S., and Tomasz, A. (1993). Possible role of a choline-containing teichoic acid in the maintenance of normal cell shape and physiology in *Streptococcus oralis*. J. Bacteriol. *175*, 1717–1722.

Kabsch, W. (2010). XDS. Acta Crystallogr. D Biol. Crystallogr. *66*, 125–132.

Korndörfer, I.P., Kanitz, A., Danzer, J., Zimmer, M., Loessner, M.J., and Skerra, A. (2008). Structural analysis of the L-alanoyl-D-glutamate endopeptidase domain of *Listeria bacteriophage* endolysin Ply500 reveals a new member of the LAS peptidase family. Acta Crystallogr. D Biol. Crystallogr. *64*, 644–650.

Korza, H.J., and Bochtler, M. (2005). Pseudomonas aeruginosa LD-carboxypeptidase, a serine peptidase with a Ser-His-Glu triad and a nucleophilic elbow. J. Biol. Chem. *280*, 40802–40812.

Koshland, D.E. (1958). Application of a theory of enzyme specificity to protein synthesis. Proc. Natl. Acad. Sci. USA *44*, 98–104.

Lovering, A.L., Safadi, S.S., and Strynadka, N.C.J. (2012). Structural perspective of peptidoglycan biosynthesis and assembly. Annu. Rev. Biochem. *81*, 451–478.

Marcyjaniak, M., Odintsov, S.G., Sabala, I., and Bochtler, M. (2004). Peptidoglycan amidase MepA is a LAS metallopeptidase. J. Biol. Chem. *279*, 43982–43989.

McCoy, A.J., Grosse-Kunstleve, R.W., Adams, P.D., Winn, M.D., Storoni, L.C., and Read, R.J. (2007). Phaser crystallographic software. J. Appl. Crystallogr. *40*, 658–674.

Metz, R., Henning, S., and Hammes, W.P. (1986). LD-carboxypeptidase activity in *Escherichia coli*. II. Isolation, purification and characterization of the enzyme from *E. coli* K 12. Arch. Microbiol. *144*, 181–186.

Meziane-Cherif, D., Stogios, P.J., Evdokimova, E., Savchenko, A., and Courvalin, P. (2014). Structural basis for the evolution of vancomycin resistance D,D-peptidases. Proc. Natl. Acad. Sci. USA *111*, 5872–5877.

Minor, W., Cymborowski, M., Otwinowski, Z., and Chruszcz, M. (2006). HKL-3000: the integration of data reduction and structure solution—from diffraction images to an initial model in minutes. Acta Crystallogr. *D62*, 859–866.

Pape, T., and Schneider, T.R. (2004). HKL2MAP: a graphical user interface for phasing with SHELX programs. J. Appl. Crystallogr. *37*, 843–844.

Royet, J., Gupta, D., and Dziarski, R. (2011). Peptidoglycan recognition proteins: modulators of the microbiome and inflammation. Nat. Rev. Immunol. *11*, 837–851.

Sauvage, E., Kerff, F., Terrak, M., Ayala, J.A., and Charlier, P. (2008). The penicillin-binding proteins: structure and role in peptidoglycan biosynthesis. FEMS Microbiol. Rev. *32*, 234–258.

Sheldrick, G.M. (2008). A short history of SHELX. Acta Crystallogr. A *64*, 112–122.

Sycuro, L.K., Pincus, Z., Gutierrez, K.D., Biboy, J., Stern, C.A., Vollmer, W., and Salama, N.R. (2010). Peptidoglycan crosslinking relaxation promotes *Helicobacter pylori*'s helical shape and stomach colonization. Cell *141*, 822–833.

Sycuro, L.K., Wyckoff, T.J., Biboy, J., Born, P., Pincus, Z., Vollmer, W., and Salama, N.R. (2012). Multiple peptidoglycan modification networks modulate *Helicobacter pylori*'s cell shape, motility, and colonization potential. PLoS Pathog. *8*, e1002603.

Sycuro, L.K., Rule, C.S., Petersen, T.W., Wyckoff, T.J., Sessler, T., Nagarkar, D.B., Khalid, F., Pincus, Z., Biboy, J., Vollmer, W., and Salama, N.R. (2013). Flow cytometry-based enrichment for cell shape mutants identifies multiple genes that influence *Helicobacter pylori* morphology. Mol. Microbiol. *90*, 869–883.

Templin, M.F., Ursinus, A., and Höltje, J.V. (1999). A defect in cell wall recycling triggers autolysis during the stationary growth phase of *Escherichia coli*. EMBO J. *18*, 4108–4117.

Vollmer, W., and Tomasz, A. (2001). Identification of the teichoic acid phosphorylcholine esterase in *Streptococcus pneumoniae*. Mol. Microbiol. *39*, 1610–1622.

Vollmer, W., Blanot, D., and de Pedro, M.A. (2008a). Peptidoglycan structure and architecture. FEMS Microbiol. Rev. *32*, 149–167.

Vollmer, W., Joris, B., Charlier, P., and Foster, S. (2008b). Bacterial peptidoglycan (murein) hydrolases. FEMS Microbiol. Rev. *32*, 259–286.

Young, F.E., and Spizizen, J. (1961). Physiological and genetic factors affecting transformation of *Bacillus subtilis*. J. Bacteriol. *81*, 823–829.

Structure, Volume *22*

Supplemental Information

Structure of the LdcB LD-carboxypeptidase Reveals the Molecular Basis of Peptidoglycan Recognition

Christopher N. Hoyland, Christine Aldridge, Robert M. Cleverley, Marie-Clémence Duchêne, George Minasov, Olena Onopriyenko, Karzan Sidiq, Peter J. Stogios, Wayne F. Anderson, Richard A. Daniel, Alexei Savchenko, Waldemar Vollmer, and Richard J. Lewis

Supplementary Figures

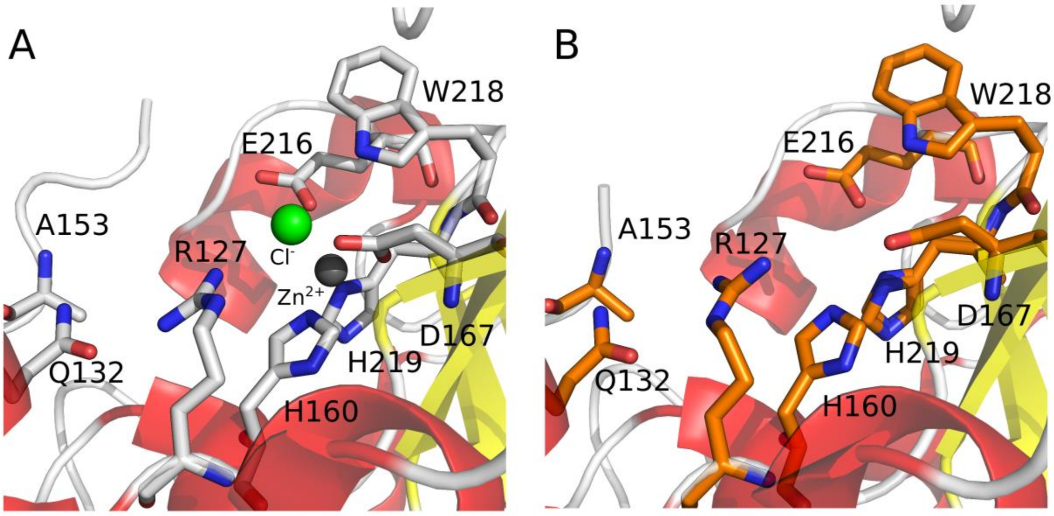

Fig. S1, related to Fig. 3. Comparison between apo and zinc-bound *Ba*LdcB.

The active sites of zinc-bound (A) and apo (B) *Ba*LdcB are shown in "stick" format, with a semi-transparent cartoon representation in the background. There are no major conformational changes in the absence of bound metal ion.

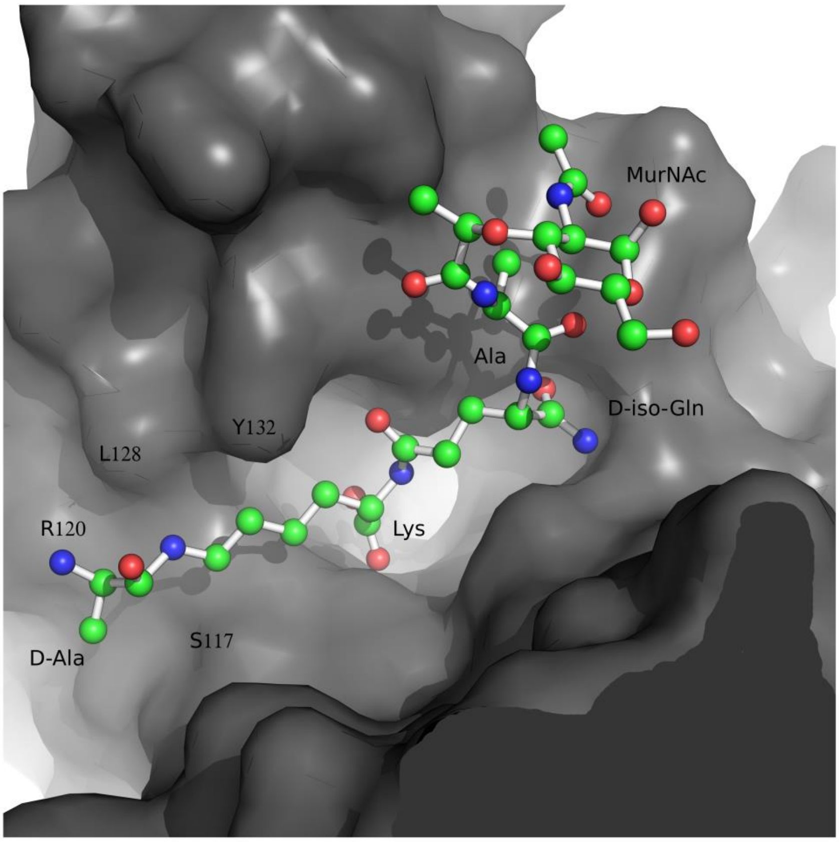

Fig. S2, related to Fig. 6. Proposed path of cross-linked cell wall peptides.

The terminal D-Asn of the ligand when bound to *Sp*LdcB occupies the S_1' subsite rather than projecting to the surface of the protein. Simple rotation of the lysyl's torsion angles presents the terminal D-aspargine to the surface of the protein to mimic the L-Lys-D-Ala (or L-Lys-Gly_n) cross-link observed between stem peptides. The path of the lysine is flanked by two highly conserved amino acids in LdcB sequences, Arg120 and Ser117, in addition to the functionally conserved Leu128. Tyr132 also flanks the lysine, but this amino acid is not conserved between sequences.

Supplementary Video

Video S1, related to Fig. 8. Conformational changes in SpLdcB on ligand binding.

Morph interpolation (UCSF Chimera) of the structural changes that occur upon ligand binding in *Sp*LdcB. α-helices are shown in red, β-strands in yellow and loops between secondary structure elements are coloured silver. The bound zinc ion is shown as a grey sphere. Key residues that move upon ligand binding are shown in stick representation and the movie was rendered in PyMOL.
